# Massive Lower Gastrointestinal Bleed caused by Typhoid Ulcer: Conservative Management

**DOI:** 10.5005/jp-journals-10018-1242

**Published:** 2017-09-29

**Authors:** Apoorv Goel, Roli Bansal

**Affiliations:** 1 Department of General Surgery, Santosh Medical College and Hospital, Ghaziabad, Uttar Pradesh, India; 2 Department of Medicine University College of Medical Sciences and Guru Teg Bahadur Hospital, Kanpur, Uttar Pradesh, India

**Keywords:** Gastrointestinal hemorrhage, Typhoid fever, Typhoid ulcer.

## Abstract

Typhoid fever is caused by gram-negative organism *Salmonella typhi.* The usual presentation is high-grade fever, but complications like gastrointestinal (GI) hemorrhage and perforation are also seen frequently. With the advent of antibiotics, these complications are rarely seen now. We present a case of a young female who was admitted with a diagnosis of typhoid fever presented with a massive GI bleed from ulcers in the terminal ileum and was managed conservatively without endotherapy and surgery.

**How to cite this article:** Goel A, Bansal R. Massive Lower Gastrointestinal Bleed caused by Typhoid Ulcer: Conservative Management. Euroasian J Hepato-Gastroenterol 2017;7(2):176-177.

## INTRODUCTION

Typhoid fever or enteric fever is caused by a gram-negative enteroinvasive organism *Salmonella typhi.^1,2^* The disease usually manifests as high-grade fever with chills and loose stools. However, GI hemorrhage and perforation is a known complication seen in the 2nd and 3rd week of the disease.^1,2^ With the advent of antibiotics, especially fluoroquinolones and third-generation cephalosporins, the rate of complications has come down. Rarely, we come across these complications but at the same time they may present in an unusual manner and may lead to diagnostic dilemmas. The usual site of ulcer formation is the terminal ileum. Bleeding if present is usually mild, which may manifest as altered blood in stools or hematochezia.^2^ The gold standard investigation for diagnosis of lower GI bleed is colonoscopy.^3,4^ Massive lower GI bleed may occur rarely and usually requires exploratory laparotomy; however, with newer advances like angioembolization and endotherapy, the rates of surgery have been decreased.^5,6^

We hereby present a case of young female from Northern India, diagnosed with typhoid fever and presented with massive lower GI bleed. Patient was managed conservatively with intensive monitoring and symptomatic management.

## CASE REPORT

**Figs 1A and B:**
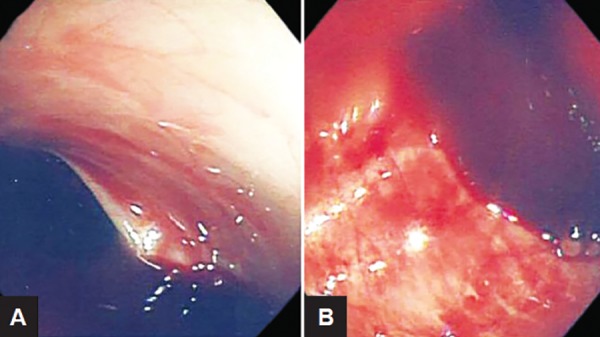
Colonoscopy of the patient

A 22-year-old married female presented with complaints of fever with chills, generalized malaise, and three episodes of loose stools of 6 days duration. She was admitted with a provisional diagnosis of enteric fever and started on injection ceftriaxone. On investigation, she was found to be anemic (hemoglobin of 9.2 mg/dL), with deranged liver function test (total bilirubin 3.3 mg/dL; direct bilirubin 2.7 mg/dL, serum glutamic oxaloacetic transaminase 215 IU/L, serum glutamic-pyruvic transaminase 299 IU/L, alkaline phosphatase 413 IU/L, total protein 6.4 g/dL, and albumin 2.8 mg/dL). The Widal test was significantly positive (H antigen was positive in a titer of 1:320 and O antigen in 1:160) and the blood culture revealed the growth of *S. typhi.* Three days after admission, she developed an increased frequency of stools with hematochezia and her hemoglobin dropped to 6.6 g/dL; however, she did not have any giddiness and loss of consciousness and was hemodynamically stable except for tachycardia (110/ min). She was given two units of packed red blood cells (PRBCs). Digital rectal examination and proctoscopy did not reveal any hemorrhoids or other local cause of lower GI bleed. She was planned for emergency colonoscopy. On colonoscopy, large bowel was filled with blood and clots and we could not proceed beyond splenic flexure (Fig. 1).

**Figs 2A and B:**
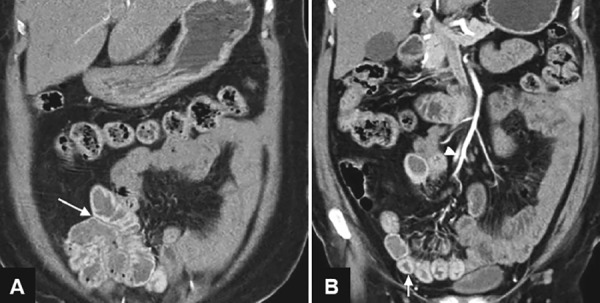
Computed tomography angiography of the patient

Since patient was losing blood, she was planned for computed tomography (CT) angiography. Meanwhile, patient’s vitals were stable except for tachycardia (110-120/min). She was also started on tablet azithromycin 1 gm daily. The CT angiography revealed bleed from a branch of ileocolic artery in the terminal ileum (Fig. 2). Patient was given another two units of PRBC. Her blood pressure was maintained and tachycardia persisted. She passed blood in stools for 2 days and gradually the frequency of stools and episodes of hematochezia decreased. Her hemoglobin came up to 9 gm/dL and tachycardia settled. She underwent check colonoscopy, seen up to cecum and terminal ileum and it revealed inflamed mucosa in terminal ileum and few ulcerations but no active bleed. She was discharged on cefixime and azithromycin for 1 week. On follow-up, patient was stable with hemoglobin of 10 gm/dL, liver function tests were normal, and there were no more episodes of hematochezia and fever.

## DISCUSSION

Typhoid fever is caused by enteroinvasive gram-negative organism *S. typhi.* The route of infection is oral ingestion of the bacteria, and contaminated water is the most common source.^1,2^ The bacteria invades the small bowel mucosa through the lymphatic and hematogenous route and further multiplies in the reticuloendothelial system. The most common and universal site is the terminal ileum due to the abundance of Peyer’s patches.^1^ Organs of reticuloendothelial system like spleen, liver, and lymph nodes are also involved. In the GI tract, apart from terminal Ileum it can also be seen in ileocecal valve, ascending colon, and transverse colon. The organism multiplies and involves the submucosa leading to ulcerations that can either perforate or erode a vessel causing the hemorrhage. These complications usually occur during the 2nd and 3rd week of illness.^2^ The incidence rate of bleeding as quoted in the literature is about 12.5%, with terminal ileum being the common site. However, the incidence has decreased with advent of newer antibiotics.

Bleeding from typhoid ulcers is usually mild, but rarely it can be massive and life threatening. It is commonly managed conservatively but in life-threatening conditions, surgical intervention in the form of right hemicolectomy or segmental resection is required.^2,4^ Now in modern era with newer advances like endotherapy and angiographic coil embolization, the need of surgical intervention has drastically reduced.^5,6^ However, these modalities are available only at a few tertiary care centers and patients like this may end up with exploratory lapa-rotomy and long-term morbidity.

This patient was managed conservatively with antibiotics as endotherapy was not possible due to nonpas-sage of colonoscope beyond splenic flexure. In previous studies over typhoid ulcer bleed it is that 90% of them are managed conservatively.^3,5^ Studies also quote that endotherapy is not as successful in typhoid ulcers when compared with peptic ulcer bleed; hence, conservative management becomes the first choice of management.^3^

However, it is uncommon to come across massive GI bleed in typhoid fever but at the same time it can be managed conservatively with antibiotics.
